# Moisture sorption isotherms and thermodynamic properties of isomaltulose‐enriched mango

**DOI:** 10.1002/jsfa.70149

**Published:** 2025-08-26

**Authors:** Juliana Rodrigues do Carmo, Jefferson Luiz Gomes Corrêa, Matheus de Souza Cruz, Maria Júlia Neves Martins, Marcio Augusto Ribeiro Sanches, Javier Telis‐Romero

**Affiliations:** ^1^ Department of Food Science Federal University of Lavras Lavras Brazil; ^2^ Department of Food Engineering and Technology São Paulo State University São José do Rio Preto Brazil

**Keywords:** ‘Tommy Atkins’, osmotic dehydration, Palatinose, isosteric heat, differential entropy

## Abstract

**Background:**

Moisture sorption isotherms and thermodynamic parameters are essential for designing and optimizing food processing and storage systems. This study aimed to evaluate these characteristics in untreated and osmotically treated mango slices, using isomaltulose and sucrose as osmotic agents. Moisture sorption isotherms were determined at temperatures ranging from 313.15 to 353.15 K using the static gravimetric method. The net isosteric heat of sorption was calculated using the Clausius–Clapeyron equation, along with differential enthalpy, entropy, and Gibbs free energy.

**Results:**

The sorption isotherms exhibited type II and III behavior. Microbiological stability was maintained at equilibrium moisture content levels below 0.20, 0.15, and 0.20 kg water per kg dry matter for untreated mango and samples treated with sucrose and isomaltulose, respectively. In all cases, equilibrium moisture content decreased with increasing temperature. Among the models tested, the Guggenheim–Anderson–de Boer (GAB) model provided the best fit to the experimental data (*R* ^2^ > 0.994, *χ* ^2^ ≤ 6.9 × 10^−4^, RMSE ≤2.6 × 10^−2^). The isosteric heat and entropy values suggested that moisture–solid interactions resembled those of pure water at moisture levels above 0.35 kg water per kg dry matter. Gibbs free energy values indicated a non‐spontaneous sorption process for untreated mango, whereas sorption in treated samples was spontaneous.

**Conclusion:**

The enthalpy–entropy compensation analysis confirmed that sorption processes in all mango samples were enthalpy‐driven. Isomaltulose‐treated mango exhibited the highest affinity for water, as evidenced by the most pronounced thermodynamic property values, highlighting its potential as a functional osmotic agent in fruit dehydration. © 2025 The Author(s). *Journal of the Science of Food and Agriculture* published by John Wiley & Sons Ltd on behalf of Society of Chemical Industry.

## INTRODUCTION

Mango (*Mangifera indica* L.) is one of the most widely consumed tropical fruits, known for its richness in macronutrients, micronutrients, and bioactive phytochemicals.^1^ Fruits such as mango can also be enhanced nutritionally through the incorporation of functional ingredients.^2^, ^3^, ^4^

One effective approach for nutritional enrichment is osmotic dehydration (OD), a process that involves immersing food in a hypertonic solution, resulting in water loss (WL) and solid gain (SG) through simultaneous isothermal mass transfer without any phase change.^5^, ^6^, ^7^ Sucrose is the most commonly used osmotic agent;^8^ however, it is rapidly digested and elicits high glycemic and insulinemic responses. In contrast, isomaltulose, commercially known as Palatinose, is a carbohydrate with a low glycemic index, is non‐cariogenic, and has potential prebiotic activity. It is recommended for the prevention and management of chronic diseases.^9^, ^10^, ^11^ Due to these characteristics, isomaltulose represents a promising alternative for improving the nutritional quality of osmotically treated products.

The hygroscopicity of fresh and dehydrated foods is closely related to their physical, chemical, and microbiological stability.^12^ The choice of solutes in OD substantially influences the composition and structure of the food matrix, influencing its moisture sorption behavior. Sorption isotherms describe the relationship between equilibrium moisture content and water activity (*a*_w_) at a constant temperature.^13^ Such information is essential for modeling and optimizing drying processes, predicting product shelf life, assessing moisture changes during storage, and selecting appropriate packaging materials.^14^

Moisture sorption behavior varies across food products, and various mathematical models have been proposed to describe these phenomena.^15^ Thermodynamic parameters – including net isosteric heat (or differential enthalpy), differential entropy, isokinetic temperature, Gibbs free energy, and the enthalpy–entropy compensation theory – can be derived from sorption isotherms obtained at multiple temperatures.^16^ These parameters provide insights into the energy requirements of moisture sorption and the nature of water–solid interactions in food matrices.

Despite the recognized benefits of isomaltulose, there is a lack of studies addressing its impact on the sorption and thermodynamic behavior of dehydrated fruits such as mango. A deeper understanding of these mechanisms is essential for designing stable, functional food products with enhanced nutritional profiles. The objective of this study was therefore to investigate the moisture sorption isotherms and related thermodynamic properties of untreated and osmotically dehydrated mango slices, using sucrose and isomaltulose as osmotic agents. The findings aim to support the development of health‐oriented dehydration strategies and improve the technical basis for industrial‐scale applications.

## MATERIAL AND METHODS

### Raw material

**Figure 1:**
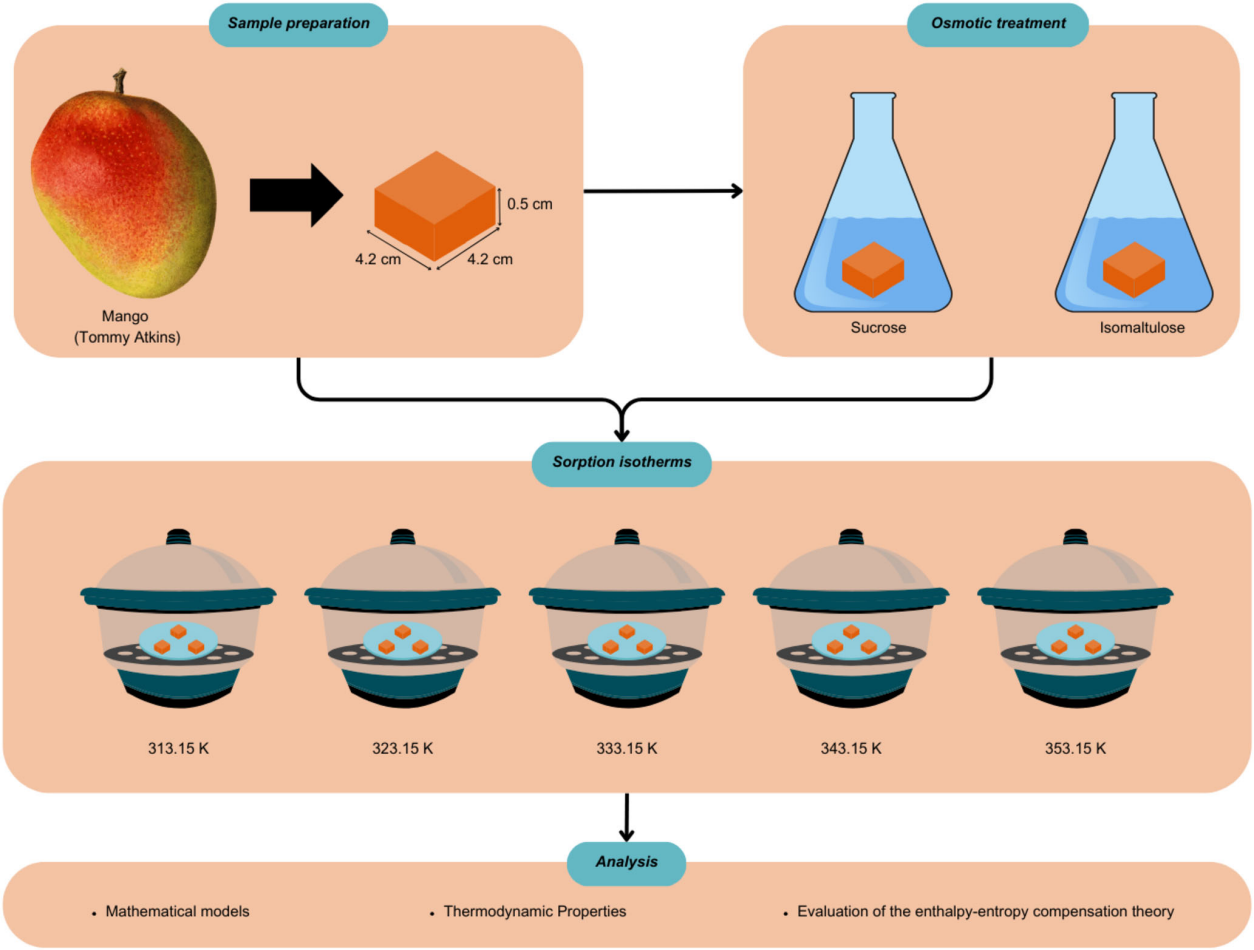
Graphical representation of the experimental planning.

Mango fruits (*Mangifera indica* L., cv. ‘Tommy Atkins’) were purchased from a local market in São José do Rio Preto, SP, Brazil (20° 49′ 11″ S, 49° 22′ 46″ W). Fruits were selected based on uniform ripeness, specifically 50% yellow peel coloration and an average soluble solid content of approximately 16 °Brix. The fruits were washed in a disinfectant solution (chlorinated water at 200 ppm) for 5 min, peeled, and had their seeds removed. The pulp was cut, using a stainless‐steel mold, into slices measuring 4.20 (±0.01) × 4.20 (±0.01) × 0.50 (±0.01) cm (length × width × thickness) (Fig. 1). The initial composition of the pulp was as follows: moisture content of 85.27 (±0.24) kg per 100 kg sample, ash 0.34% (±0.01), lipids 0.22% (±0.01), proteins 0.90% (±0.01), total carbohydrates 11.17% (±0.23), and dietary fiber 2.10% (±0.02). These values were consistent with the literature.^1^, ^17^

### Preparation of osmotic solutions

Two saturated osmotic solutions were prepared using distilled water: one with analytical‐grade sucrose and the other with isomaltulose (commercially known as Palatinose, Sigma‐Aldrich, Steinheim, Germany). Solutes were weighed on an analytical balance (AUX220, Shimadzu, Kyoto, Japan; precision 0.1 mg). Saturation at 313.15 K was determined according to data from Martins *et al*.^18^ and Carmo *et al*.^3^, ^9^, ^19^ to ensure complete solubilization, the solutions were stirred using a magnetic stirrer (C‐MAG HS 7, IKA, USA), with temperature monitored *via* a thermocouple (AK05, AKASO, Sao Leopoldo, Brazil). Saturation was confirmed by measuring the soluble solids content using a portable digital refractometer (HI 86801, Hanna Instruments, Tambore Barueri, Brazil). Although sucrose exhibits higher solubility than isomaltulose, both solutions presented comparable *a*_w_ values.^19^

### Osmotic treatment

Mango slices were immersed in glass containers containing one of the osmotic solutions at 313.15 ± 1.10 K and atmospheric pressure (1017 hPa) for 24 h. A solution‐to‐sample ratio of 20:1 (ww^−1^) was used to prevent dilution of the osmotic medium.^20^ The process temperature was controlled using a thermostatic water bath (MA‐184, Marconi, Piracicaba, Brazil). After treatment, the samples were rinsed in distilled water at 276.15 ± 1.00 K for 30 s to stop dehydration and remove excess surface solute,^21^ then dried with absorbent paper.^22^ The final moisture contents were 58.60 (±0.01) and 62.2 (±0.49) kg per 100 kg sample for sucrose and isomaltulose treatments, respectively. Water loss (WL) was 31.05% and 26.96%, whereas solid gain (SG) was 9.32% and 9.98% for isomaltulose‐ and sucrose‐treated samples, respectively.

### Moisture sorption isotherms

Moisture sorption isotherms were determined for untreated and osmotically dehydrated mango samples at five temperatures: 313.15, 323.15, 333.15, 343.15, and 353.15 K, using the gravimetric‐static method. Temperature control was ensured using a thermostatic chamber (MA415, Marconi, Brazil) for 313.15 K, and an oven (MA030, Marconi) for the other temperatures. Glass desiccators containing saturated salt solutions were used to establish different relative humidity levels. Table 1 lists the corresponding *a*_w_ values for each salt and temperature. Samples (2.0000 ± 0.0001 g) were weighed in triplicate and placed in suspended containers to avoid contact with the salt solution. All experiments were conducted under atmospheric pressure. Moisture content before and after equilibrium was determined using AOAC methods.^23^ The equilibrium data were used to construct the sorption isotherms.

**Table 1:** Water activity (*a*_w_) of saturated salt solutions at different temperatures

Salt	*a*_w_				
	Temperature (K)				
	313.15	323.15	333.15	343.15	353.15
LiBr	0.0580	0.0553	0.0533	0.0523	0.0520
LiCl	0.1121	0.1110	0.1095	0.1075	0.1051
LiI	0.1455	0.1238	0.0998	0.0723	‐
MgCl_2_	0.3160	0.3054	0.2926	0.2777	0.2605
NaI	0.3288	0.2921	0.2595	0.2357	0.2252
Mg(NO_3_)_2_	0.4842	0.4544	‐	‐	‐
NaBr	0.5317	0.5093	0.4966	0.4970	0.5143
KI	0.6609	0.6449	0.6311	0.6193	0.6097
NaCl	0.7468	0.7443	0.7450	0.7506	0.7629
(NH_4_)_2_SO_4_	0.7991	0.7920	‐	‐	‐
KCl	0.8232	0.8120	0.8025	0.7949	0.7890
KNO_3_	0.8903	0.8478	‐	‐	‐
K_2_SO_4_	0.9641	0.9582	‐	‐	‐

### Sorption isotherm modeling

The experimental data were fitted to theoretical, empirical, and semi‐empirical models listed in Table 2. The quality of fit was evaluated using the coefficient of determination (*R* ^2^), reduced chi‐squared (*χ* ^2^), and root mean square error (RMSE).

**Table 2:** Sorption isotherm models

Model	Equation	References
GAB	$x_{\mathrm{eq}}=\frac{X_{m}\cdot C\cdot k\cdot a_{w}}{\left(1-k\cdot a_{w}\right)\cdot \left(1+\left(c-1\right)\cdot k\cdot a_{w}\right)}$	Van den Berg^52^
Peleg	$x_{\mathrm{eq}}=k_{1}\cdot a_{w}^{n_{1}}+k_{2}\cdot a_{w}^{n_{2}}$	Peleg^53^
Oswin	$x_{\mathrm{eq}}=M\cdot {\left(\frac{a_{w}}{1-a_{w}\;}\right)}^{N}$	Oswin^54^
Henderson	$x_{\mathrm{eq}}={\left(-\frac{1}{H_{1}\;}\cdot \ln\;\left(1-a_{w}\;\right)\right)}^{\frac{1}{H_{2}}}$	Boquet^55^
Halsey	$x_{\mathrm{eq}}={\left(-h_{1}\cdot \ln\;\left(a_{w}\right)\right)}^{-\frac{1}{h_{2}}}$	Halsey^56^

*Note*: *x*_*e*q_ is the equilibrium moisture content; *a*_w_ is the water activity; *X*_m_ is the monolayer moisture content – dry basis; *C*, *k*, *k*_1_, *k*_2_, *n*_1_, *n*_2_, *M*, *N*, *H*_1_, *H*_2_, *h*_1_ and *h*_2_ are empirical parameters of the sorption models.

### Thermodynamic properties

#### Isosteric heat of sorption (*q*_st_)

The net isosteric heat of sorption (*q*_st_, J mol^−1^) was determined using the integrated form of the Clausius–Clapeyron equation, Eqn (1), based on the slope of the linear relationship between the natural logarithm of water activity (*ln*(*a*_*w*_)) and the inverse of absolute temperature (1/*T*) at constant equilibrium moisture content (*Xeq*):^24^

(1) $${\left[\frac{d\left(\ln\;a_{w}\;\right)}{d\left(1/T\right)}\right]}_{X_{\mathrm{eq}}}=\frac{-q_{\mathrm{st}}}{R}$$

where *a*_w_ is the water activity (dimensionless), *T* is the absolute temperature (K), *R* is the universal gas constant (8.314 J/mol K), and *qst* is the isosteric heat of sorption.

#### Differential entropy and Gibbs free energy

The differential entropy of sorption (*Δ*S, J mol K^−1^) was estimated using the Gibbs–Helmholtz equation, Eqn (2).^25^ The Gibbs free energy (*Δ*G, J mol^−1^) was calculated using the van't Hoff equation, Eqn (3). Equation (4) was obtained by combining both equations, allowing the determination of *Δ*S from the slope (*Δ*S/R) of the linear relationship between ln(*a*_w_) and 1/T at a constant equilibrium moisture content (*X*_eq_):

(2) $$\mathrm{\Delta G}=q_{\mathrm{st}}-\mathrm{\Delta ST}$$

(3) $$\mathrm{\Delta G}=-\mathrm{RT}\;\ln\;a_{w}$$

(4) $$\ln\;a_{w}=-\frac{q_{\mathrm{st}}}{\mathrm{RT}}+\frac{\mathrm{\Delta S}}{R}$$

### Enthalpy–entropy compensation theory

The enthalpy–entropy compensation theory was evaluated using Eqn (5). This approach is applicable when the isokinetic temperature (*T*_*B*_, K) differs from the harmonic mean temperature (*T*_*hm*_, K), which is calculated using Eqn (6). According to Krug *et al*.,^26^ if *TB* > *Thm*, the sorption process is considered enthalpy driven; conversely, if *T*_*B*_ < *T*_*hm*_, the process is entropy driven.

(5) $$\mathrm{\Delta H}=T_{B}\mathrm{\Delta S}+\Delta G_{B}$$

(6) $$T_{\mathrm{hm}}=\frac{n}{\sum_{1}^{n}\frac{1}{T}}$$

where *ΔG*_B_ is Gibbs free energy at *T*_B_ (J mol^−1^), n is the number of temperatures at which the isotherms were obtained.

The isokinetic temperature (*T*_*B*_) and its statistical confidence interval at a confidence level of (1–*α*)100% were calculated using Eqns 7, 8 and 9:

(7) $$T_{B}=T_{B}\;\pm \;t_{m-2,\alpha /2\sqrt{\mathrm{Var}\left(T_{B}\right)}}$$

where

(8) $$T_{B}=\frac{\sum \left(\mathrm{\Delta H}-\underline{\mathrm{\Delta H}}\right)\left(\mathrm{\Delta S}-\underline{\mathrm{\Delta S}}\right)}{\sum {\left(\mathrm{\Delta S}-\underline{\mathrm{\Delta S}}\right)}^{2}}$$

(9) $$\mathrm{Var}\left(T_{B}\right)=\frac{\sum {\left(\mathrm{\Delta H}-\underline{\Delta G_{B}}-T_{B}\mathrm{\Delta S}\right)}^{2}}{\sum \left(m-2\right)\sum {\left(\mathrm{\Delta S}-\underline{\mathrm{\Delta S}}\right)}^{2}}$$

where *m* is the number of data pairs (*ΔH*, *ΔS*), $\underline{∆S}$ is the mean differential entropy and $\underline{\mathrm{\Delta H}}$ is the mean differential enthalpy.

### Statistical analysis

Chemical composition results were expressed as means ± standard deviations (*n* = 3). Sorption models were fitted by non‐linear regression using OriginPro 8.0 (OriginLab Corp., Northampton, MA, USA). Models were considered satisfactory when *R* ^2^ values were close to 1, and χ^2^ and RMSE values were minimized.

## RESULTS AND DISCUSSION

### Moisture sorption isotherms

**Figure 2:**
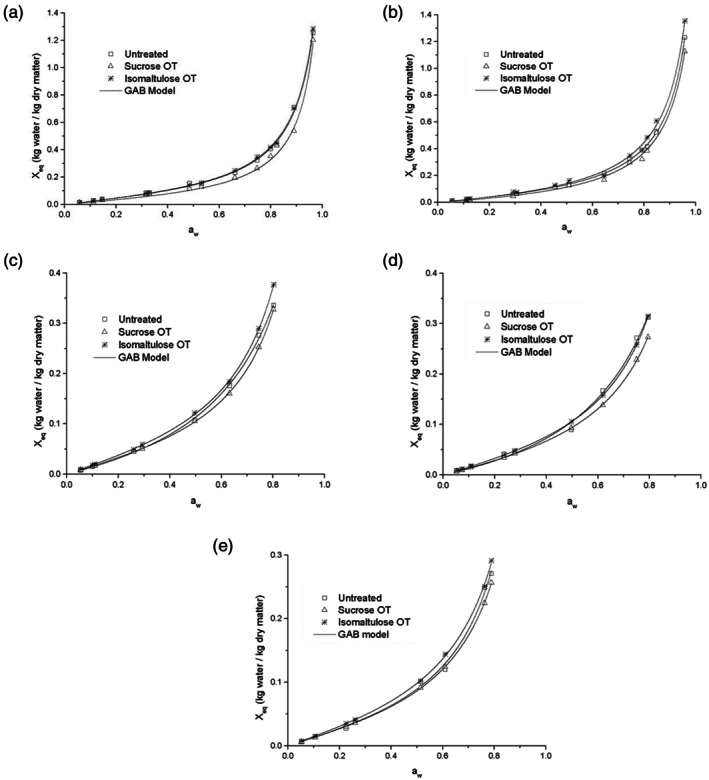
Sorption isotherms fitted to the Guggenheim–Anderson–de Boer (GAB) model of untreated and osmodehydrated mangos with sucrose and isomaltulose at drying temperatures of (a) 313.15; (b) 323.15; (c) 333.15; (d) 343.15, and (e) 353.15 K.

Figure 2 presents the moisture adsorption isotherms of untreated and osmotically treated mango slices at different temperatures. In general, mango slices treated with sucrose exhibited lower equilibrium moisture contents than those treated with isomaltulose or the untreated samples. At temperatures ranging from 313.15 to 353.15 K, the equilibrium moisture content varied from 1.255 to 0.271 kg water per kg dry matter for untreated mango, from 1.203 to 0.257 kg water per kg dry matter for sucrose‐treated samples, and from 1.283 to 0.291 kg water per kg dry matter for isomaltulose‐treated samples. These results indicate that isomaltulose‐treated mango was more hygroscopic than the sucrose‐treated samples. According to Udomkun *et al*.,^27^ the type of carbohydrate present in the matrix influences sorption behavior due to variations in solubility, availability of binding sites, and the binding energy of molecular structures. A similar trend was reported by Falade and Awoyele,^28^ who observed greater hygroscopicity in bananas subjected to sucrose‐based osmotic dehydration in comparison with untreated samples.

Based on the classification criteria proposed by Yanniotis and Blahovec,^29^ the isotherms of untreated mango generally exhibited type II behavior, characteristic of solution‐like materials. For osmotic treatment (OT) samples, type II behavior was maintained up to 323.15 K. However, at temperatures above 333.15 K, the isotherms shifted to a type‐III pattern. Both type II and III isotherms have also been reported for other osmotically treated products.^30^, ^31^, ^32^

As illustrated in Fig. 2, a sharp increase in moisture content occurred at *a*_*w*_ > 0.60, which was consistent with the behavior of high‐sugar food systems. This phenomenon may be attributed to the dissolution of crystalline sugars at lower *a*_*w*_ levels and their transformation into amorphous structures at higher *a*_*w*_.^33^ This behavior highlights the need for careful handling and storage of these products in environments with relative humidity above 60%. Under such conditions, packaging materials with low water vapor permeability are recommended.^14^ Furthermore, given the presence of bioactive compounds such as ascorbic acid, carotenoids, and phenolic compounds known to be highly susceptible to oxidation packaging should also be opaque and impermeable to air and light.^34^

For both untreated and OT mangoes, equilibrium moisture content decreased with increasing temperature at constant *a*_*w*_. This is explained by the higher kinetic energy of water molecules at elevated temperatures, which reduces the strength of intermolecular interactions and the ability of water to bind to active sites in the food matrix.^35^ At lower temperatures, reduced molecular mobility allows for stronger water binding, particularly to macromolecules such as carbohydrates and proteins. This behavior has also been observed in other osmotically treated fruits.^13^, ^36^

According to the Guggenheim–Anderson–de Boer (GAB) model (Table 2), monolayer moisture content (*X*_*m*_) values ranged from 0.07 to 0.12 kg water per kg dry matter for untreated mango, from 0.07 to 0.13 for sucrose‐treated mango, and from 0.08 to 0.13 for isomaltulose‐treated mango (Table 3). As expected, *X*_*m*_ values decreased with increasing temperature. This temperature dependency has been associated with the reduction in the number of available sorption sites due to physicochemical changes in the food matrix.^37^ This behavior has been documented in a variety of foods, including mango subjected to sucrose osmotic treatment.^32^, ^38^

**Table 3:** Predicted parameters of fitted models for sorption isotherms of untreated mango at different temperatures

*T* (K)	GAB					
	*X*_m_	*C*	*K*	*R* ^2^	*χ* ^2^	RMSE
313.15	0.1169	2.0000	0.9453	0.998	2.7·10^−4^	1.6·10^−2^
323.15	0.1158	1.6214	0.9510	0.999	4.2·10^−5^	6.5·10^−3^
333.15	0.0897	1.6990	0.9802	0.999	2.1·10^−5^	4.6·10^−3^
343.15	0.0960	1.4598	0.9488	0.997	3.8·10^−5^	6.2·10^−3^
353.15	0.0694	1.8862	0.9893	0.995	5.1·10^−5^	7.1·10^−3^

	Peleg						RMSE
	*k*_1_	*n*_1_	*k*_2_	*n*_2_	*R* ^2^	*χ* ^2^	
313.15	0.3533	1.2211	1.3431	10.4226	0.999	1.1·10–4	8.9·10^−3^
323.15	1.3661	11.4232	0.4195	1.5951	0.999	8.3·10^−5^	7.6·10^−3^
333.15	0.1655	1.0023	0.5258	4.2777	0.999	4.2·10^−6^	1.5·10^−3^
343.15	0.1114	0.8197	0.5366	3.8097	0.999	4.5·10^−5^	5.0·10^−3^
353.15	0.1971	1.2165	0.6969	7.1636	0.997	5.5·10^−5^	5.2·10^−3^

	Oswin				
	*M*	*N*	*R* ^2^	*χ* ^2^	RMSE
313.15	0.1698	0.6152	0.990	1.3·10^−3^	3.6·10^−2^
323.15	0.1532	0.6693	0.995	5.3·10^−4^	2.3·10^−2^
333.15	0.1115	0.8055	0.997	3.9·10^−5^	6.2·10^−2^
343.15	0.1047	0.8288	0.995	7.5·10^−5^	8.6·10^−2^
353.15	0.0889	0.8566	0.996	4.3·10^−5^	6.6·10^−2^

	Henderson				
	*H*_1_	*H*_2_	*R* ^2^	*χ* ^2^	RMSE
313.15	2.8603	0.6791	0.994	7.3·10^−4^	2.7·10^−2^
323.15	2.7979	0.6348	0.997	3.5·10^−4^	1.9·10^−2^
333.15	3.8688	0.7969	0.998	3.1·10^−5^	5.5·10^−3^
343.15	3.8767	0.7740	0.994	7.9·10^−5^	8.9·10^−3^
353.15	4.1343	0.7466	0.992	8.8·10^−5^	9.4·10^−4^

	Halsey				
	*h*_1_	*h*_2_	*R* ^2^	*χ* ^2^	RMSE
313.15	0.0537	1.4688	0.978	2.8·10^−3^	5.3·10^−2^
323.15	0.0581	1.3465	0.984	1.8·10^−3^	4.2·10^−2^
333.15	0.0824	0.9412	0.986	2.0·10^−4^	1.4·10^−2^
343.15	0.0832	0.9107	0.986	1.9·10^−4^	1.4·10^−2^
353.15	0.0763	0.8889	0.992	9.2·10^−5^	9.6·10^−3^

*Note*: GAB is Guggenheim–Anderson–de Boer; *T* is temperature; *X*_m_ is the monolayer moisture content – dry basis; *C*, *k*, *k*_1_, *k*_2_, *n*_1_, *n*_2_, *M*, *N*, *H*_1_, *H*_2_, *h*_1_, and *h*_2_ are the constants of the model; *R* ^2^ is the coefficient of determination; RMSE is the root mean square error; *χ* ^2^ is reduced chi‐squared.

According to Labuza,^39^ foods with *Xm* ≤ 0.10 kg water per kg dry matter are considered microbiologically stable. In this study, samples stored at 333.15 K or above met this criterion. The monolayer moisture content is considered a critical indicator for product stability, as it represents the optimal moisture level to minimize physical and chemical degradation during storage.^32^

#### Modelling of sorption isotherms

Among the evaluated models, the Peleg and GAB models demonstrated good predictive performance for describing the moisture sorption behavior of both untreated and osmotically treated mango samples (Tables 3, 4, and 5). The GAB model was selected for further analysis due to its strong theoretical foundation, physical significance of its parameters, and its use of fewer constants compared to the Peleg model. Figure 2 presents the sorption isotherms fitted using the GAB model.

**Table 4:** Predicted parameters of fitted models for sorption isotherms of osmodehydrated mango with sucrose at different temperatures

*T* (K)	GAB					
	*X*_m_	*C*	*K*	*R* ^2^	χ^2^	RMSE
313.15	0.0861	2.0000	0.9646	0.994	6.9·10^−4^	2.6·10^−2^
323.15	0.1150	1.1990	0.9457	0.996	4.2·10^−4^	2.1·10^−2^
333.15	0.1064	1.4789	0.9130	0.999	7.2·10^−6^	2.7·10^−3^
343.15	0.0858	1.5974	0.9344	0.999	9.4·10^−7^	9.7·10^−4^
353.15	0.0690	1.9085	0.9724	0.999	8.8·10^−7^	9.4·10^−4^

	Peleg						
	*k*_1_	*n*_1_	*k*_2_	*n*_2_	*R* ^2^	*χ* ^2^	RMSE
313.15	0.4739	1.8421	1.4502	17.7766	0.994	8.0·10^−4^	2.4·10^−2^
323.15	1.2924	9.3678	0.2755	1.2599	0.998	2.8·10^−4^	1.4·10^−2^
333.15	0.2266	1.1919	0.6837	6.7801	0.998	2.3·10^−6^	1.1·10^−3^
343.15	0.1766	1.1226	0.4641	5.3283	0.998	5.4·10^−7^	5.0·10^−4^
353.15	0.2030	1.2607	0.6416	7.6267	0.998	2.1·10^−6^	1.0·10^−3^

	Oswin				
	*M*	*N*	*R* ^2^	*χ* ^2^	RMSE
313.15	0.1355	0.6653	0.996	4.2·10^−4^	2.1·10^−2^
323.15	0.1384	0.6743	0.990	1.0·10^−3^	3.2·10^−2^
333.15	0.1045	0.8182	0.999	3.5·10^−6^	1.9·10^−3^
343.15	0.0926	0.8071	0.999	5.7·10^−6^	2.4·10^−3^
353.15	0.0859	0.8276	0.999	1.5·10^−6^	1.2·10^−3^

	Henderson				
	*H*_1_	*H*_2_	*R* ^2^	*χ* ^2^	RMSE
313.15	3.0071	0.6153	0.990	1.1·10^−3^	3.4·10^−2^
323.15	2.9537	0.6280	0.994	6.1·10^−4^	2.5·10^−2^
333.15	3.9650	0.7844	0.996	5.7·10^−5^	7.6·10^−3^
343.15	4.5475	0.8045	0.998	2.2·10^−5^	4.6·10^−3^
353.15	4.5667	0.7793	0.997	3.2·10^−5^	5.6·10^−3^

	Halsey				
	*h*_1_	*h*_2_	*R* ^2^	*χ* ^2^	RMSE
313.15	0.0481	1.3684	0.989	1.2·10^−3^	3.4·10^−2^
323.15	0.0517	1.3379	0.978	2.1·10^−3^	4.6·10^−2^
333.15	0.0801	0.9274	0.993	9.9·10^−5^	9.9·10^−3^
343.15	0.0710	0.9283	0.991	9.5·10^−5^	9.7·10^−3^
353.15	0.0691	0.9148	0.994	5.7·10^−5^	7.6·10^−3^

*Note*: GAB is Guggenheim–Anderson–de Boer; *T* is the temperature; *X*_m_ is the monolayer moisture content – dry basis; *C*, *k*, *k*_1_, *k*_2_, *n*_1_, *n*_2_, *M*, *N*, *H*_1_, *H*_2_, *h*_1_ and *h*_2_ are the constants of the model; *R* ^2^ is the coefficient of determination; RMSE is the root mean square error; χ^2^ is reduced chi‐squared.

**Table 5:** Predicted parameters of fitted models for sorption isotherms of osmodehydrated mango with isomaltulose at different temperatures

*T* (K)	GAB					
	*X*_m_	*C*	*K*	*R* ^2^	*χ* ^2^	RMSE
313.15	0.1220	1.9393	0.9433	0.999	1.2·10^−4^	1.1·10^−2^
323.15	0.1325	1.3900	0.9486	0.997	4.6·10^−4^	2.2·10^−2^
333.15	0.0973	1.8924	0.9671	0.999	3.5·10^−6^	1.9·10^−3^
343.15	0.0904	1.7551	0.9523	0.999	5.2·10^−9^	7.2·10^−5^
353.15	0.0790	1.9083	0.9684	0.999	7.6·10^−6^	2.8·10^−3^

	Peleg						
	*k*_1_	*n*_1_	*k*_2_	*n*_2_	*R* ^2^	*χ* ^2^	RMSE
313.15	0.4555	1.5478	1.3268	12.1160	0.999	1.4·10^−4^	9.8·10^−3^
323.15	0.3145	1.1647	1.5655	9.2297	0.999	2.4·10^−4^	1.3·10^−2^
333.15	0.2615	1.2030	0.7813	6.7768	0.999	4.7·10^−6^	1.6·10^−3^
343.15	0.2174	1.1701	0.5880	6.0248	0.999	2.0·10^−6^	1.1·10^−3^
35 315	0.2413	1.2940	0.8020	8.3366	0.999	1.3·10^−5^	2.6·10^−3^

	Oswin				
	*M*	*N*	*R* ^2^	*χ* ^2^	RMSE
313.15	0.1723	0.6169	0.992	1.1·10^−3^	3.3·10^−2^
323.15	0.1675	0.6718	0.991	1.2·10^−3^	3.5·10^−2^
333.15	0.1196	0.8206	0.999	5.0·10^−6^	2.2·10^−3^
343.15	0.1050	0.8140	0.999	3.1·10^−6^	1.8·10^−3^
353.15	0.0976	0.8215	0.999	7.3·10^−6^	2.7·10^−3^

	Henderson				
	*H*_1_	*H*_2_	*R* ^2^	*χ* ^2^	RMSE
313.15	2.8236	0.6795	0.997	4.5·10^−4^	2.1·10^−2^
323.15	2.6311	0.6298	0.994	8.1·10^−4^	2.9·10^−2^
333.15	3.5467	0.7815	0.996	7.3·10^−5^	8.5·10^−3^
343.15	4.0439	0.7966	0.997	3.5·10^−5^	5.9·10^−3^
353.15	4.1979	0.7864	0.996	4.3·10^−5^	6.6·10^−3^

	Halsey				
	*h*_1_	*h*_2_	*R* ^2^	*χ* ^2^	RMSE
313.15	0.0552	1.4639	0.979	2.7·10^−3^	5.2·10^−2^
323.15	0.0662	1.3427	0.981	2.7·10^−3^	5.2·10^−2^
333.15	0.0913	0.9251	0.993	1.3·10^−4^	1.2·10^−2^
343.15	0.0810	0.9214	0.992	1.1·10^−4^	1.0·10^−2^
353.15	0.0766	0.9204	0.993	8.2·10^−5^	9.0·10^−3^

*Note*: GAB is Guggenheim–Anderson–de Boer; *T* is the temperature; *X*_m_ is the monolayer moisture content – dry basis; *C*, *k*, *k*_1_, *k*_2_, *n*_1_, *n*_2_, *M*, *N*, *H*_1_, *H*_2_, *h*_1_, and *h*_2_ are the constants of the model; *R* ^2^ is the coefficient of determination; RMSE is the root mean square error, and *χ* ^2^ is reduced chi‐squared.

The superior performance of the GAB model in representing sorption phenomena in biological materials is well documented in the literature.^30^, ^38^, ^40^ Its widespread use is attributed to its ability to describe the sorption behavior accurately over a broad range of water activity levels, making it a reliable tool for predicting moisture dynamics in food systems.

### Thermodynamic properties

Thermodynamic properties were evaluated for equilibrium moisture content ranging from 0.15 to 0.60 kg water per kg dry matter at all tested temperatures, using *a*_*w*_ values predicted by the GAB model.

#### Isosteric heat of sorption and differential entropy

**Figure 3:**
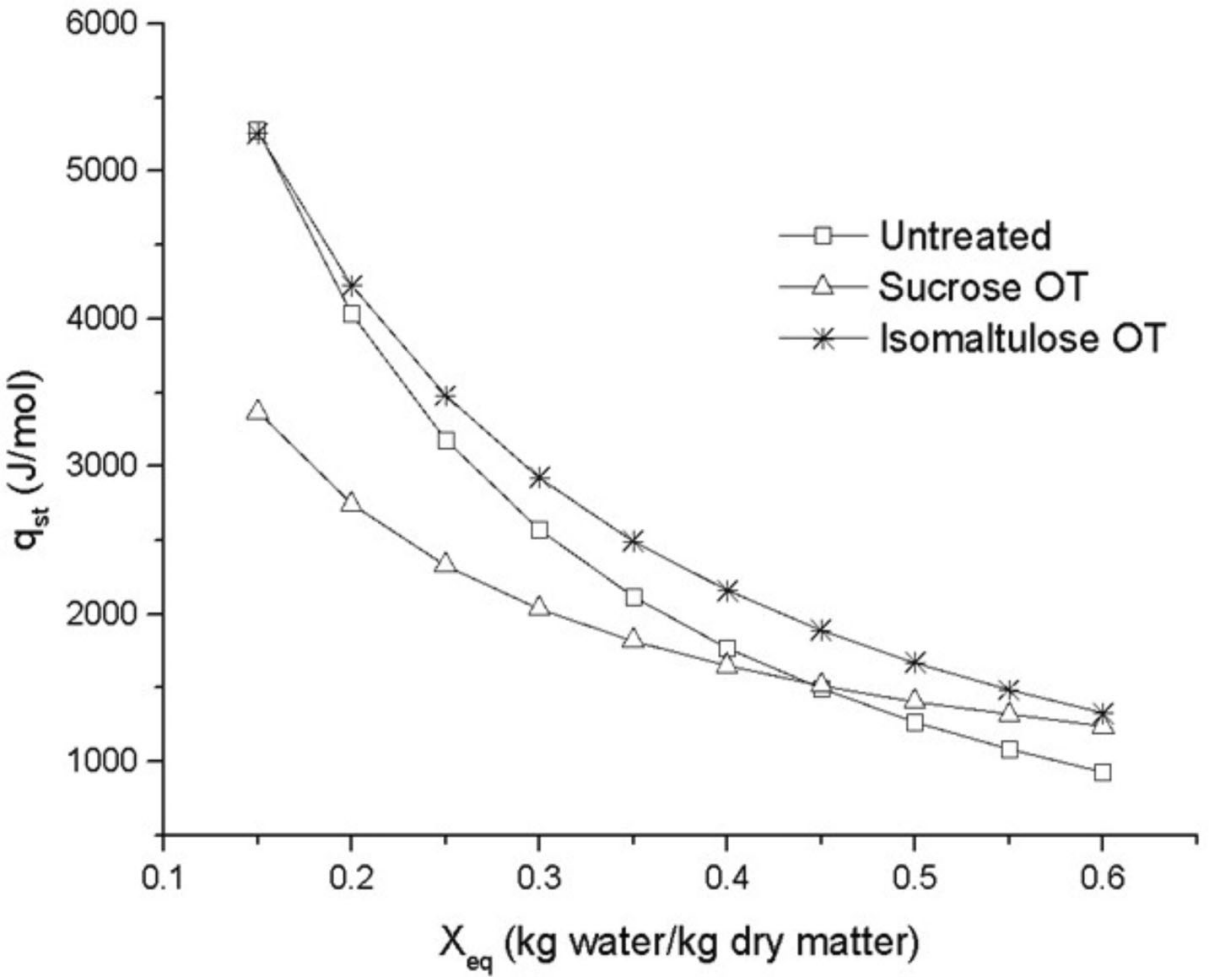
Liquid isosteric sorption heat for the untreated mango and the osmodehydrated mango with sucrose and isomaltulose as a function of equilibrium moisture content.

**Figure 4:**
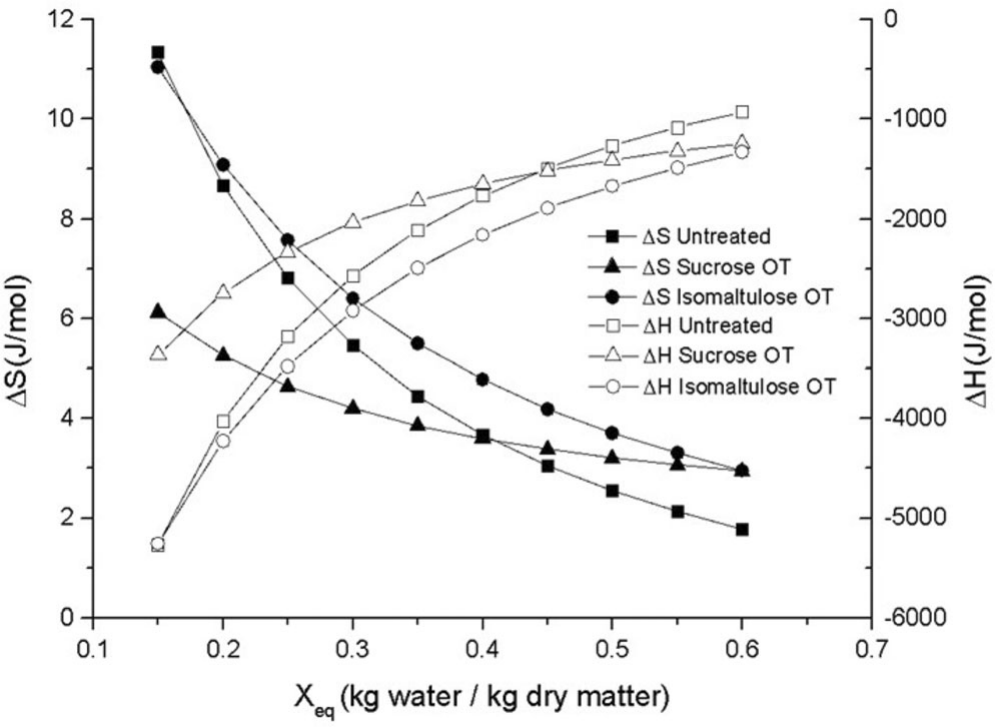
Change in enthalpy and entropy as a function of equilibrium moisture contents.

Figures 3 and 4 illustrate the behavior of net isosteric heat of sorption (*q*_st_) and differential entropy (*Δ*S) as functions of moisture content for untreated and osmotically treated mango samples. At moisture content levels below 0.35 kg water per kg dry matter, *q*_st_ increased exponentially as moisture decreased. This trend was especially pronounced in isomaltulose‐treated samples, which exhibited a higher affinity for water, indicating a greater moisture‐retention capacity compared to untreated samples. Similar behavior was reported by Paes *et al*.^41^ in osmotically treated *cambuci* fruit using sorbitol as the osmotic agent.

As shown in Fig. 3, *q*_st_ values ranged from 928.09 to 5279.47 J mol^−1^ for untreated mango, from 1240.20 to 3365.76 J mol^−1^ for sucrose‐treated samples, and from 1328.83 to 5258.44 J mol^−1^ for isomaltulose‐treated samples. These findings are consistent with studies on green bananas,^42^ papaya seeds,^43^ *cambuci*,^41^ quince,^44^ and papaya.^27^ At around 0.45 kg water per kg dry matter, the curves for untreated and sucrose‐treated mango intersected, indicating a shift in sorption behavior.

Higher isosteric heat values at low moisture contents reflect stronger binding of water to the food matrix, particularly to macromolecules such as carbohydrates, fibers, and proteins. As moisture increases, the energy required for sorption decreases, and water begins to exhibit properties similar to those of free or bulk water.^45^

Enthalpy (*ΔH*) showed a similar pattern to *q*_st_, representing the total energy required for sorption. As Fig. 4 shows, *ΔH* values were negative (−928.09 to −5279.47 J mol^−1^), confirming the exothermic nature of the sorption process and the presence of attractive forces between water molecules and the mango matrix.^46^

Differential entropy (*Δ*S) reflects the degree of order or disorder in the sorption system and is associated with the arrangement of water molecules and their interaction with the food matrix. In this study, *Δ*S ranged from 1.78 to 11.33 J mol K^−1^ for untreated samples, 2.94 to 6.12 J mol K^−1^ for sucrose‐treated samples, and 2.96 to 11.04 J/mol K for isomaltulose‐treated samples (Fig. 4). The observed decrease in *ΔS* with increasing moisture content is consistent with a progressive occupation of high‐energy sorption sites.^47^, ^48^

At low moisture levels, the high *ΔS* values suggest restricted molecular mobility, as water binds strongly to available sites. As moisture increases, lower‐energy sites are occupied, requiring less energy for sorption. This also facilitates multilayer formation, where water molecules are more mobile. As Fig. 4 shows, higher entropy values at low moisture contents indicate increased energy demand for binding water under these conditions.^24^

#### Gibbs free energy

Gibbs free energy represents the energy available to perform useful work in a system.^24^ McMinn *et al*.^46^ pointed out that the sign of *Δ*G can indicate whether a process is spontaneous (*ΔG* < 0) or non‐spontaneous (*ΔG* > 0). In this study, *ΔG* was calculated based on the thermodynamic relationships presented earlier, and the isokinetic temperature (*T*_B_) was determined from the linear regression of Eqn (8).

For untreated mango, a positive *ΔG* value of 106.96 J mol^−1^ was observed, indicating a non‐spontaneous sorption process. In contrast, the osmotically treated samples exhibited negative *ΔG* values: −727.69 J mol^−1^ for sucrose‐treated and −131.59 J mol^−1^ for isomaltulose‐treated mango. These results suggest that the sorption process was thermodynamically favorable (spontaneous) in the osmotically treated samples, likely due to structural changes in the matrix and enhanced binding affinity induced by the osmotic agents.

#### Theory of enthalpy–entropy compensation

**Figure 5:**
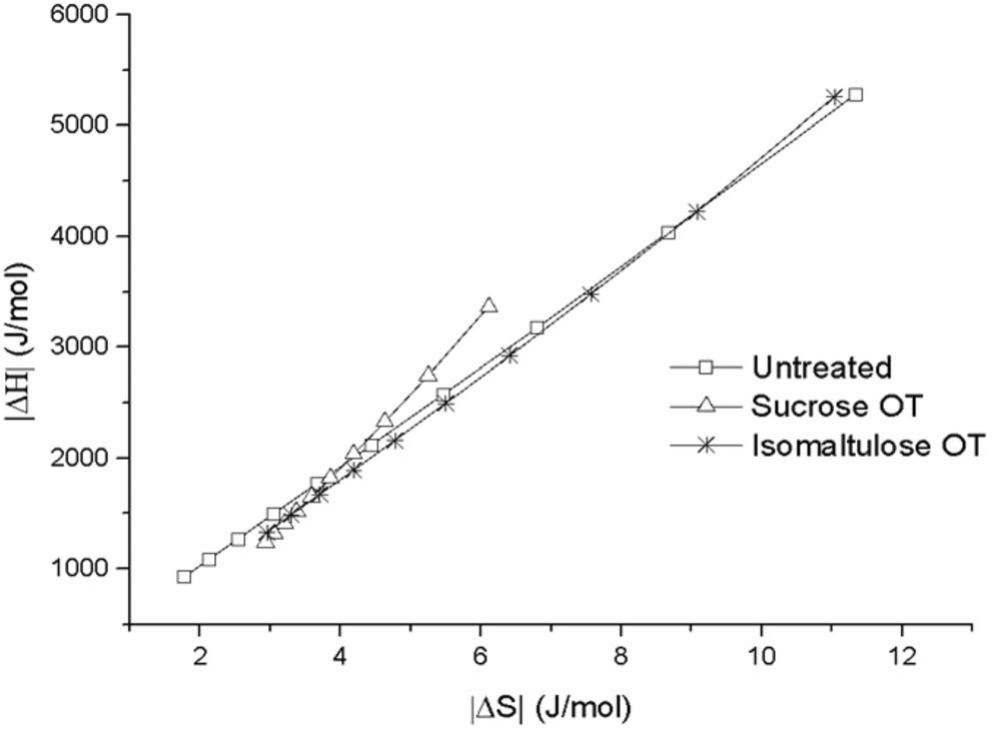
Linear regression of differential enthalpy at differential entropy values.

The enthalpy–entropy compensation theory was evaluated by analyzing the linear relationship between differential enthalpy (*ΔH*) and differential entropy (*ΔS*). As Fig. 5 shows, a strong linear correlation was observed for both untreated and osmotically treated mango samples (*R* ^2^ ≥ 0.995), confirming the existence of a compensation effect within the studied moisture content range. This relationship indicates that changes in *ΔH* are systematically accompanied by changes in *ΔS* and, consequently, in *ΔG*.^49^

The compensation phenomenon was confirmed by the inequality between *T*_*B*_ and *T*_*hm*_, as proposed by Krug *et al*.^26^, ^50^ In this study, *T*_*hm*_ was 332.55 K, and *T*_*B*_ values were 454.14 ± 3.73 K for untreated mango, 663.57 ± 20.41 K for sucrose‐treated mango, and 482.36 ± 34.61 K for isomaltulose‐treated mango. As *T*_*B*_ was greater than *T*_*hm*_ in all cases, it can be concluded that the moisture sorption processes in these samples were enthalpy‐controlled.^51^

This result aligns with previous findings reported by Noshad *et al*.,^44^ who also observed enthalpy‐driven adsorption during osmotic dehydration of quince using sucrose solutions in the temperature range of 303.15–333.15 K.

## CONCLUSIONS

Untreated and osmotically treated mango samples using sucrose and isomaltulose exhibited type II and type III moisture sorption isotherms. Microbiological stability was achieved at equilibrium moisture content below 0.20, 0.15, and 0.20 kg water per kg dry matter for untreated mango, and OT mango treated with sucrose and isomaltulose, respectively. These results indicate that untreated mango and isomaltulose‐treated samples required more stringent storage conditions than those treated with sucrose. A typical inverse relationship between temperature and equilibrium moisture content was observed across all samples.

The GAB model accurately described the sorption behavior and was effective in fitting the experimental data. Thermodynamic analysis revealed that enthalpy and entropy changes reflected the nature of water–matrix interactions, which resembled those of pure water at moisture levels above 0.35 kg water per kg dry matter. Gibbs free energy values indicated that moisture sorption was non‐spontaneous in untreated mango, while it occurred spontaneously in OT samples. Enthalpy–entropy compensation analysis further demonstrated that the sorption process in all cases was enthalpy‐driven.

## CONFLICT OF INTEREST

The authors declare no competing interests.

## FUNDING INFORMATION

This work was financially supported by the Coordenação de Aperfeiçoamento de Pessoal de Nível Superior (CAPES) (code 001 and grant 88887.468140/2019‐00), the Conselho Nacional de Desenvolvimento Científico e Tecnológico (CNPq) (project number 166378/2018‐6 and project number 314191/2021‐6) and the Fundação de Amparo à Pesquisa do Estado de Minas Gerais (FAPEMIG).

## AUTHOR CONTRIBUTIONS

**Juliana Rodrigues do Carmo:** investigation, methodology, data curation, formal analysis, validation, visualization, writing, review, and editing. **Jefferson Luiz Gomes Corrêa:** conceptualization, funding acquisition, supervision, and review. **Matheus de Souza Cruz:** investigation, data curation, visualization, writing, and review. **Maria Júlia Neves Martins:** investigation, methodology, data curation, and review. **Marcio Augusto Ribeiro Sanches:** investigation, methodology, data curation, and review. **Javier Telis‐Romero:** investigation, methodology, data curation, and review.

## Data Availability

Data will be made available on request.
